# Nestin is essential for cellular redox homeostasis and gastric cancer metastasis through the mediation of the Keap1–Nrf2 axis

**DOI:** 10.1186/s12935-021-02184-4

**Published:** 2021-11-12

**Authors:** Jing Lv, Meiqiang Xie, Shufen Zhao, Wensheng Qiu, Shasha Wang, Manming Cao

**Affiliations:** 1 Department of Oncology, The Affiliated Hospital of Qingdao University/Key Laboratory of Cancer Molecular and Translational Research, The Affiliated Hospital of Qingdao University, No.16 Jiangsu Road, Qingdao, 266000 Shandong China; 2 Medical Oncology, Central People’s Hospital of Zhanjiang, Zhanjiang, 524000 Guangdong China; 3 Department of Medical Oncology, Zhujiang Hospital, Southern Medical University, No.253 Gongye Road, Haizhu District, Guangzhou, 510282 Guangdong China

**Keywords:** Gastric cancer, Nestin, Keap1, Nrf2, Oxidative stress, Apoptosis, Metastasis

## Abstract

**Background:**

Gastric cancer (GC) is a common malignancy of the digestive system. Antioxidant activity is regarded as a possible mechanism in ectopic cancer. Hence, oxidative stress regulation is being evaluated for cancer treatment. Previous research has demonstrated that Nestin is associated with antioxidative resistance via its modulation of the Kelch-like ECH-associated protein 1 (Keap1)–nuclear factor erythroid 2-related factor 2 (Nrf2) pathway.

**Methods:**

We determined the role of Nestin-mediated redox homeostasis and tumor phenotypes in GC cells.

**Results:**

We found that the Nestin expression level was high in GC tissues and cell lines. Nestin knockdown in the GC cell lines SGC-7901 and MKN-45 reduced viability, induced apoptosis, decreased antioxidant enzyme generation, and repressed GC metastasis. Nestin binds to Keap1, resulting in Nrf2 degradation and influencing downstream gene expression. Nestin knockdown resulted in the downregulation of Nrf2 expression in GC cells. The restoration of Nrf2 expression or treatment with the Nrf2 activator sulforaphane counteracted the inhibitory effect of Nestin knockdown on the proliferation, migration, invasion, and antioxidant enzyme production in GC cells. Moreover, xenograft GC tumors exhibited a slower growth rate than those of the control group in vivo.

**Conclusions:**

Taken together, these findings suggest that the Nestin–Keap1–Nrf2 axis confers oxidative stress resistance and plays an important role in the proliferation, migration, and invasion of GC cells.

## Background

Gastric cancer (GC) is the fourth most common cancer and the second leading cause of cancer-related mortality worldwide [1]. The development of GC involves many stages and underlying factors, and the identification of the GC subtypes will provide a direction for patient stratification and targeted therapy development [2, 3]. Studies have indicated that different molecule or protein expression profiles may be related to varying prognoses [4]. Four molecular subtypes, i.e., Epstein–Barr virus, microsatellite instable, genomically stable, and chromosomal instability, were recently correlated with the different patterns of molecular changes, disease development, and prognosis via gene expression analysis [2, 3]. Previous research has indicated that the release and removal of reactive oxygen species (ROS), including superoxide (O2^•−^), hydroxide (•OH^−^), and hydrogen peroxide (H_2_O_2_), are imbalanced, resulting in moderate oxidative stress [5]. Cancer cells instinctively produce higher levels of ROS than healthy cells owing to mitochondrial or metabolic dysfunction [6, 7]; therefore, developing effective antioxidant defenses that can regulate ROS to suitable levels to prevent cancer occurrence and transformation is warranted [8]. Hence, targeting the antioxidant capacities of GC cells might show a positive treatment effect.

Oxidative stress is an imbalance between oxidant production and antioxidant activity. The microenvironment is characterized by different stress conditions, including hypoxia and nutrient deprivation, induced by defective tumor vasculature or genotoxic and oxidative stress caused by rapid cell division or therapy [9]. Patients with GC experience high levels of oxidative stress, which contribute to the progression of GC. Oxidative stress participates in GC progression by affecting critical effector expression [10]. ROS are activated factors in gastric carcinogenesis in both humans and mice [10]. Oxidative stress leads to cell membrane, protein, and DNA damage [11] and contributes to cell apoptosis by regulating p38α mitogen-activated protein kinases (MAPK) [12]. Excessive ROS in cells can damage tissues, leading to tumorigenesis, particularly in the gastrointestinal tract. Therefore, many regulatory factors have been targeted to influence intracellular antioxidant defenses to maintain oxidant homeostasis in cancer cells. The transcription factor nuclear factor erythroid 2-related factor 2 (Nrf2) regulates the expression levels of various antioxidant enzymes, including glutathione S-transferase and NAD(P)H quinone dehydrogenase 1 (NQO1), by binding with enhancer sequences called antioxidant response elements (AREs) [13, 14]. Constitutive Nrf2 stabilization and activation are correlated with unfavorable patient prognosis in many types of human cancers, including bladder cancer and lung cancer [14]. Nrf2 activity is strictly inhibited by its binding with the Kelch-like ECH-associated protein 1 (Keap1)–Cullin 3 (Cul3) E3-Rbx1 ligase complex, which restricts its transfer from the cytosol to the nucleus. Consequently, constitutive Nrf2 expression is involved in maintaining basal antioxidant levels [14]. Mechanistic research has suggested that Keap1 acts as a major scaffold in E3 ubiquitin ligase with Cul3.

Nestin, an intermediate filament protein, is a more specific marker for freshly formed blood vessels and a treatment target owing to its ability to inhibit angiogenesis [15]. Nestin acts as a specific marker for angiogenesis in malignancies, particularly in colorectal carcinomas [16] and prostate cancers [17]. The expression level of Nestin in the microvessel density is proposed to be a more sensitive marker of longer survival compared with the expression level of CD34 [18]; however, in GC, no association between Nestin-positive microvessel density and patients’ clinical results has been demonstrated. Wang et al. showed that Nestin competitively binds to the Kelch domain to shield Nrf2 from Keap1-mediated degradation, thereby increasing the expression levels of antioxidant enzymes. Nestin binds directly with both Keap1 and Nrf2 and upregulates Nrf2 expression to modulate oxidative equilibrium in lung cancer [19]. This study determined the effect of Nestin on in vivo tumor formation and in vitro tumor phenotypes (proliferation and metastasis) during GC development and the underlying mechanism. The involvement of the Keap1–Nrf2 axis in the Nestin-modulated antioxidant response and tumor phenotypes (proliferation and metastasis) of GC was also elucidated. The study findings indicated that the Nestin–Keap1–Nrf2 pathway serves as a target for suppressing malignant GC phenotypes, including proliferation and metastasis.

## Methods

### Ethical statement

All experiments performed in this work were approved by the Ethics Committee of Zhujiang Hospital, Southern Medical University, and all participants provided informed consent before participation. Animal experiments were approved by the Animal Ethics Committee of Zhujiang Hospital, Southern Medical University. Appropriate measures were taken to reduce animal suffering as far as possible.

### Study participants

Twenty-two patients with GC and nine healthy volunteers from Zhujiang Hospital, Southern Medical University were enrolled in this study. All patients (mean age, 55.5 ± 10.9 years) underwent surgery, and the pathological diagnosis of GC was confirmed. The patients neither received drug treatments before surgery nor exhibited any signs of distant metastasis. Gastric tissue samples were obtained from nine healthy volunteers. The samples were isolated and instantly frozen at − 80 °C. Sample diagnoses were performed by two independent pathologists.

### Cell culture

Human GC cells, SGC-7901, BGC-823, MKN-28, NCI-N87, and MKN-45, as well as GES-1 cells, were provided by Shanghai Institutes for Biological Sciences, Chinese Academy of Sciences. These cells were routinely cultured in high-glucose Dulbecco’s modified Eagle’s medium (11965084, Gibco™) for SGC-7901 or Roswell Park Memorial Institute-1640 (11875093, Gibco™) for other cell lines. The media were supplemented with fetal bovine serum (FBS) (10%, 16140071, Gibco™), streptomycin (100 µg/ml, 10378016, Gibco™), and penicillin (100 U/ml, 10378016, Gibco™). Cultures were incubated in a damp condition at 37 °C under 5% CO_2_. Cells at the exponential growth phase were used.

### Vectors and transfection

pSM2-encoding shRNAs were provided by Open Biosystems (Huntsville, AL, USA). Flag-Nrf2 overexpression vectors were constructed in a pcDNA3 vector, and a pcDNA3 empty vector served as a negative control. Myc-Keap1 overexpression vectors were constructed in a pCMV vector, and a pCMV empty vector served as a negative control. The cells were seeded in 6-well plates at a density of 5 × 10^5^ cells/well and then transfected with 200 ng shRNA-NC, 200 ng shRNA-Nestin, 500 ng pcDNA3 empty vector, or 500 ng pcDNA3-Flag-Nestin using Lipofectamine 2000 (Invitrogen) until the cells attained 80% confluency. The medium was changed 6 h after transfection, and the cells were collected 1.5–2 d after transfection.

### Quantitative polymerase chain reaction (qPCR)

Total RNA was extracted from GC cells and tissue samples (100 mg) using TRIzol reagent (Invitrogen, USA), and the RNA concentration and quality were measured using Nanodrop2000. cDNA was produced using reverse transcription using Oligo (dT) 20 primer and the MMLV First-Strand Kit (Invitrogen, USA) for qPCR. Gene expressions were determined using qPCR with the relevant kits, and all procedures were performed in accordance with the manufacturer’s instructions. The reaction conditions were as follows: denaturation at 95 °C for 10 min, followed by 40 cycles of denaturation at 95 °C for 15 s, and extension at 60 °C for 40 s. The expression levels of target mRNAs were determined using the 2^−ΔΔCT^ method with glyceraldehyde 3-phosphate dehydrogenase mRNA as the internal reference. All experiments were performed in triplicate.

### Western blot (WB) analysis

Cells were lysed using radioimmunoprecipitation assay buffer (pH 8.0) supplemented with protease inhibitor cocktail (Roche Applied Science). Protein concentrations were determined using a bicinchoninic acid assay kit. The proteins were separated using sodium dodecyl sulfate–polyacrylamide gel electrophoresis and transferred onto polyvinylidene difluoride membranes (Millipore, MA, USA). The vacant sites were blocked using 4% bovine serum albumin. The membrane were then incubated with the primary antibodies overnight at 4 °C and then rinsed with TBST. Then, the membrane was incubated with the secondary antibodies for 1 h at room temperature. After rinsing the membrane with TBST a few times, bands on the membrane were developed using the Maximum Sensitivity Substrate Kit (Thermo, MA, USA).

### Cell viability

Cell viability was evaluated using the Cell Counting Kit-8 (CCK-8) assay according to the manufacturer’s instructions. Cells were seeded in 96-well plates, CCK-8 reagent (10 μl) was added, and cells were incubated at 37 °C for 2 h. Optical density at 450 nm was measured using the Infinite M200 plate reader (Tecan, Switzerland).

### Bromodeoxyuridine (BrdU) staining assay

Cell proliferation was further determined via a BrdU assay kit (Roche Diagnostics GmbH, Germany). Cells were seeded in 6-well plates (2 × 10^5^ cells/well) on disinfected coverslips. BrdU solution (10 μM) was added 3 days later, and the cells were incubated for 5 h. BrdU integration was monitored by adding anti-BrdU-POD monoclonal antibody (100 μl) and further incubation at room temperature for 0.5 h. Subsequently, peroxidase substrate (100 μl) with substrate enhancer was added, and cells were incubated at room temperature for an additional 15 min. Finally, immune complexes were detected by measuring the optical density at 490 nm.

### Flow cytometry (FC)

Apoptosis was evaluated using the Annexin V-FITC Apoptosis Detection Kit (Invitrogen, Carlsbad, CA, USA). Pulmonary microvascular endothelial cells were digested and rinsed with cold phosphate-buffered saline. The cells (1 × 10^6^ cells/ml) were resuspended in binding buffer (100 μl) containing Annexin V. Then, propidium iodide (PI) was added, and cells were cultured at room temperature for 20 min in the dark. The number of apoptotic cells was determined using a flow cytometer (BD Biosciences, San Jose, CA, USA).

### Determination of antioxidant activity

The glutathione (GSH) content, total antioxidant activity, and catalase (CAT) and superoxide dismutase (SOD) activities were determined using the relevant assay kits.

### Luciferase assay

ARE was cloned into a pGL3-basic luciferase reporter plasmid. Cells (1 × 10^5^ cells/well) were seeded into 12-well plates in triplicate. After incubation for 1 d, the cells were treated with ARE luciferase reporter plasmids (200 ng) using a transfection reagent (OriGene). Cells were then recovered in a medium containing 10% FBS for 1 d. Then, 2 d after transfection, firefly and *Renilla* luciferase signals were determined and expressed as the increase in activation relative to the reporter alone.

### Transwell invasion assay

Cells were collected through trypsinization and rinsed with D-Hanks solution once. To determine cell invasion, 8-μm pore size Matrigel inserts (200 μg/ml) were placed in 24-well plates. F-12 (400 μl) was added to the lower chamber with FBS (10%) and HGF (20 ng/ml). Next, cells (1 × 10^5^) were seeded on the upper chamber. After incubation for 20 h, the cells that migrated through the pores were stained with crystal violet and observed through a microscope. Six fields with a magnification of 4 × were randomly selected for counting the migrated cell numbers.

### Wound healing assay

Confluent cells were scratched using a 10-μl pipette tip. The cells were allowed to migrate into the wound for 36 h and subsequently fixed. The scratched area was observed under a microscope. The migration ratio (%) was calculated as follows: width_36 h_/width_0 h_.

### Detection of antioxidant capacity

The GSH content, SOD activity, and CAT activity were measured using GSH-Glo Glutathione Assay kit (Promega), SOD Assay kit (Sigma-Aldrich), and CAT Activity Assay kit (Biovision), respectively, according to the manufacturers’ instructions.

### Xenograft tumors in nude mice

Twenty-four clean-grade female BALB/c nude mice (age, 28–42 d; weight, 20 ± 2 g) were purchased from the Animal Experimental Center of Zhujiang Hospital, Southern Medical University. Mice were anesthetized with sodium pentobartital (50 mg/kg with 33 IU heparin i.p.). SGC-7901 cells transfected with shRNA-NC or shRNA-Nestin were resuspended in 50% Matrigel (BD Biosciences, Bedford, MA). The cell concentration was subsequently adjusted to 1 × 10^7^ cells/ml. The left axilla of each mouse was transfected with a subcutaneous injection of single-cell suspension (0.2 ml, 5 × 10^6^ cells), and each group included eight mice. At day 28, the mice were euthanized and the tumor weight and size as well as lymph node metastasis were evaluated.

### Statistical analyses

All data were expressed as means ± standard deviation. Student’s t-test and analysis of variance were used to analyze differences among multiple groups and between two groups, respectively. P < 0.05 indicated a significant difference.

## Results

### Nestin expression in GC tissues and cells

**Fig. 1:**
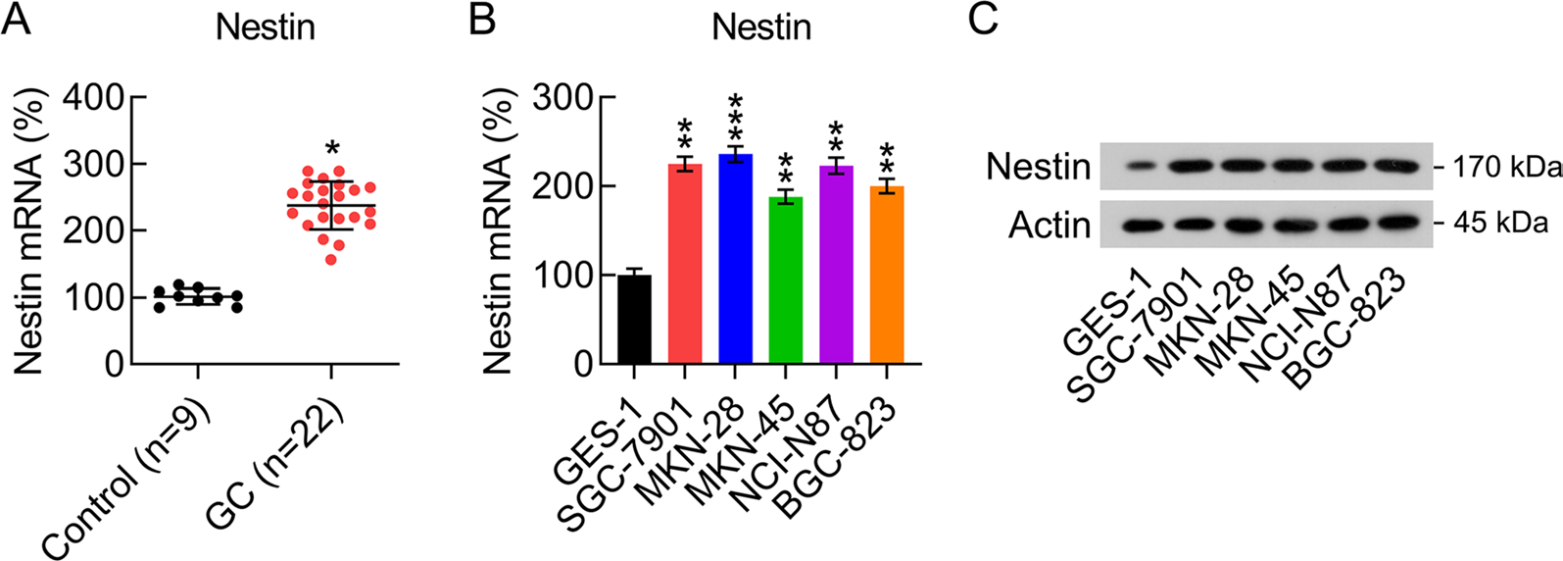
Nestin expression in GC tissues and cells. **A** qPCR analysis was performed to show Nestin expression level in GC tissues (n = 22) and normal gastric tissues (n = 9). **B** qPCR and **C** WB revealed Nestin expression levels in GC cells and normal gastric GES-1 cells (n = 3). The results are expressed as means ± SD. *P < 0.05, **P < 0.01 vs. the indicated groups

To determine the role of Nestin during GC development, we examined its expression using qPCR in 22 GC tissues and 9 healthy gastric tissues. Nestin expression was upregulated in the GC tissues compared with that in healthy tissues (Fig. 1A). In addition, the mRNA and protein expression of Nestin was upregulated in GC cells compared with that in GES-1 cells, as assessed using qPCR and WB (Fig. 1B, C); these results suggest that nestin plays a role in GC development.

### Effect of Nestin knockdown on GC cell viability, antioxidant capacity, and metastasis

**Fig. 2:**
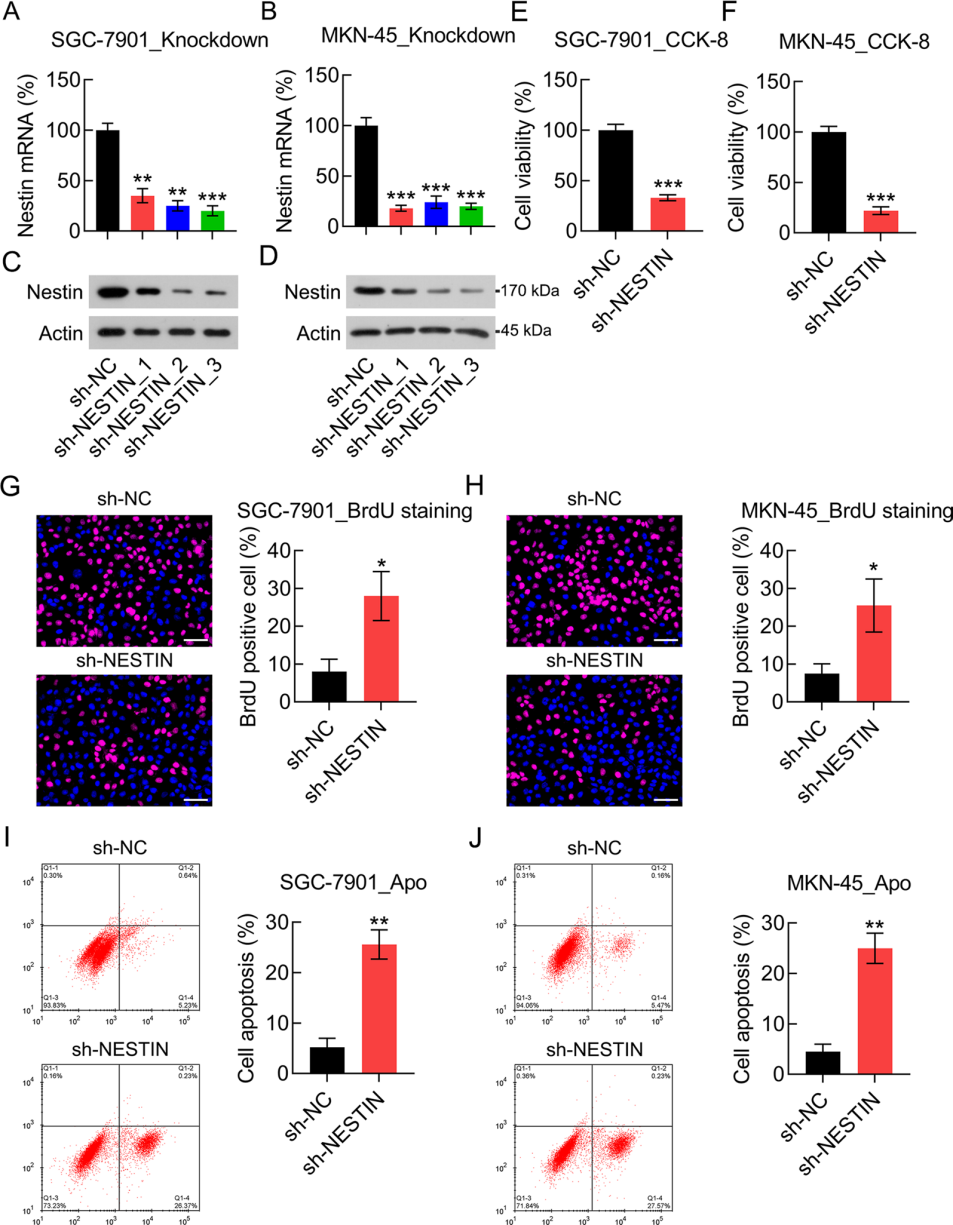
Effect of Nestin knockdown on GC cell viability and apoptosis. GC cells were transfected with shRNA-NC and shRNA-Nestin-1, -2, and -3 for 36 h. **A**, **B** qPCR and **C**, **D** WB were performed to determine Nestin mRNA and protein expression level in GC cells, respectively. **E**, **F** The CCK-8 assay showed the viability of GC cells 2 d after transfection. **G**, **H** The bromodeoxyuridine (BrdU) assay showed the viability of GC cells 2 d after transfection. Scale bar, 50 μm. **I**, **J** Annexin V-FITC/PI FC showed the percentage of apoptotic cells 2 d after transfection (n = 3). The results are expressed as means ± SD. *P < 0.05, **P < 0.01, ***P < 0.001 vs. the indicated groups

To investigate the association between the tumor phenotypes of GC cells and Nestin, Nestin-short hairpin RNAs (shNestin1, sh-Nestin2, and sh-Nestin3) were used to knockdown Nestin in GC cells (Fig. 2A–D). The CCK-8 assay was then performed to examine the effect of Nestin knockdown on the viability of GC cells, and the results indicated that cell viability was significantly reduced after Nestin knockdown (Fig.2E, F). Furthermore, the BrdU incorporation assay showed that nestin knockdown decreased the proliferation rate of GC cells (Fig. 2G, H). Annexin V/PI FC was performed to determine the influence of Nestin knockdown on GC cell apoptosis. Nestin knockdown increased the apoptosis rate of GC cells (Fig. 2I, J). These findings suggest that Nestin knockdown reduces cell viability and proliferation by inducing the apoptosis of GC cells.

**Fig. 3:**
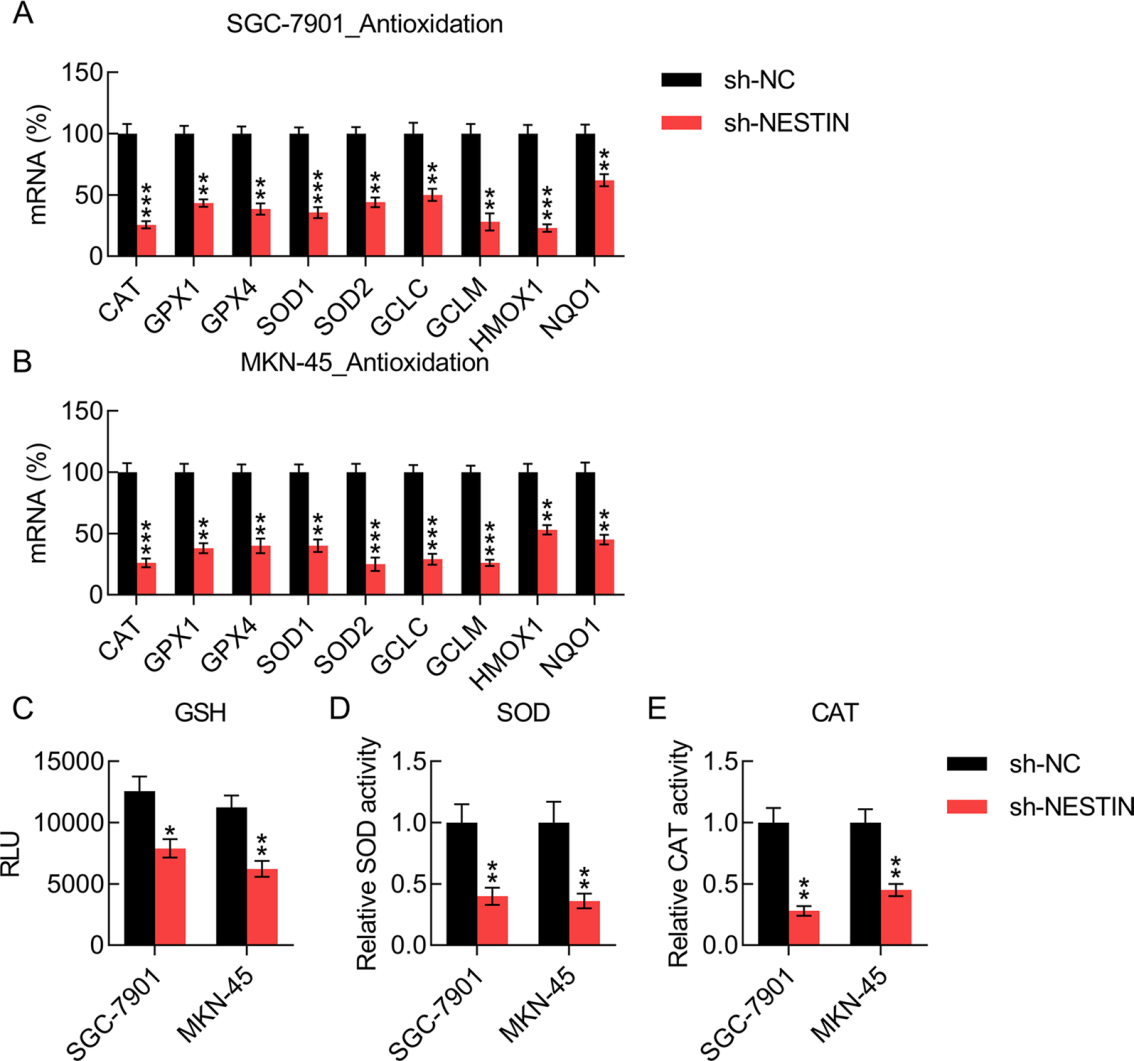
Effect of Nestin knockdown on GC cell antioxidant capacity. GC cells were transfected with shRNA-NC and shRNA-Nestin for 36 h. **A**, **B** qPCR revealed the expression of antioxidant-related genes in both Nestin knockdown and control groups. (C, D, E) GSH levels, SOD activity, and CAT levels were examined in GC cells under different transfection (n = 3). The results are expressed as means ± SD. *P < 0.05, **P < 0.01, ***P < 0.001 vs. the indicated groups

It was reported that patients with GC experience high levels of oxidative stress, which contribute to GC progression. Oxidative stress participates in GC progression by affecting critical effector expression [10]. To investigate whether Nestin affects the antioxidant activity of GC cells, the gene expression levels of different antioxidant proteins, including glutamate–cysteine ligase (GCL), GCL modifier subunit (GCLM), SOD1, SOD2, glutathione peroxidase 1 (GPX1), GPX4, CAT, NQO1, and heme oxygenase 1, were examined [15]. The results suggested that Nestin knockdown downregulates the mRNA levels of these proteins in GC cells (Fig. 3A, B). In addition, the antioxidant levels as well as GSH, SOD, and CAT activities were decreased (Fig. 3C–E). These findings indicate that Nestin is essential for the total antioxidant activity in GC cells.

**Fig. 4:**
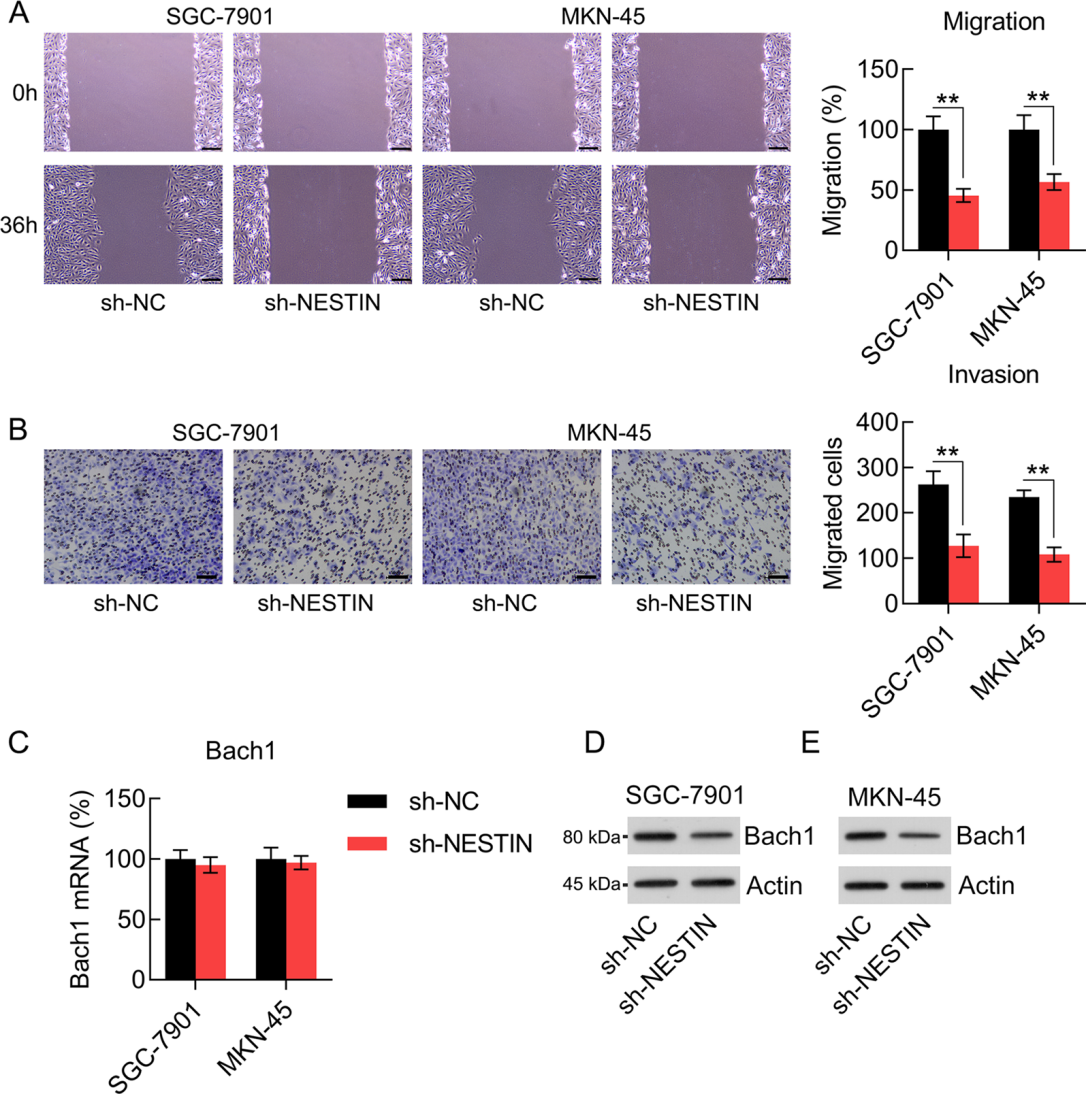
Effect of Nestin knockdown on GC cell migration and invasion abilities. GC cells were transfected with shRNA-NC and shRNA-Nestin for 36 h. **A** The migration and **B** invasion abilities of GC cells were detected using wound healing assays and Transwell invasion assay. Scale bar, 50 μm. **C** qPCR and **D**, **E** WB analysis were used to determine the Bach1 mRNA and protein expression levels in GC cells, respectively, (n = 3). The results are expressed as means ± SD. **P < 0.01 vs. the indicated groups

To determine the influence of Nestin on the migration and invasion abilities of GC cells, cells were subjected to the wound healing and Transwell migration assays. The wound healing assay showed that Nestin knockdown significantly decreased the migration rate of GC cells (Fig. 4A). Meanwhile, Nestin knockdown also inhibited GC cell invasion ability, as detected using the Transwell migration assay (Fig. 4B). A previous study demonstrated that Nrf2 leads to Bach1 accumulation in lung cancer and promotes lung cancer metastasis in a Bach1-dependent manner [20]. Therefore, we determined the Bach1 expression level in GC cells with or without Nestin knockdown. Our results showed that the mRNA expression level of Nestin and Bach1 was not altered in each transfection group. However, we observed that the protein expression level of Nestin and Bach1 significantly decreased after Nestin knockdown (Fig. 4C–E). These data suggest that Nestin knockdown influences the migration and invasion of GC cells.

### Nestin expression is associated with the activation of the Keap1–Nrf2 pathway

**Fig. 5:**
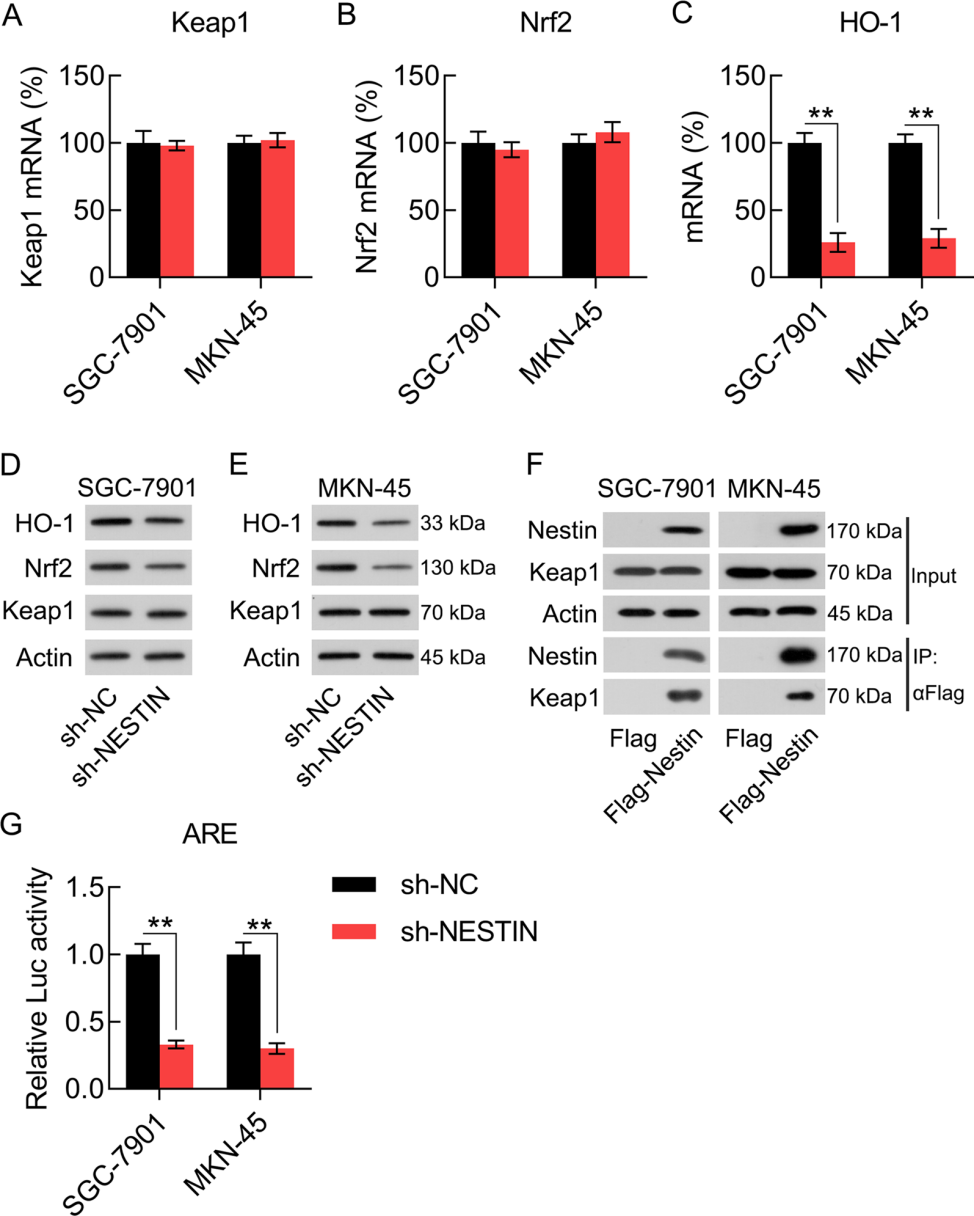
Nestin protects Nrf2 expression in GC cells. GC cells were transfected with shRNA-NC and shRNA-Nestin for 36 h. **A**–**C** qPCR revealed the effect of Nestin knockdown on the mRNA expression levels of Keap1, Nrf2, and HO-1 in GC cells. **D**, **E** WB analysis revealed the effect of Nestin knockdown on the protein expression levels of Keap1, Nrf2, and HO-1 in GC cells. **F** Nestin directly binds with Keap1. Flag-Nestin and myc-Keap1 were transfected into GC cells. Whole-cell lysates were used for immunoprecipitation using anti-Flag antibodies, and the proteins obtained were blotted with the indicator antibodies. **G** GC cells with Nestin knockdown were exposed to ARE luciferase reporter. The luciferase activity was determined 36 h after transfection (n = 3). The results are expressed as means ± SD. **P < 0.01 vs. the indicated groups

The Keap1–Nrf2 axis is generally regarded as a major transcription factor that modulates antioxidant defense systems. The present study investigated whether Nestin affects the expression of antioxidant proteins by modulating the Keap1–Nrf2 pathway. First, the mRNA expression levels of Keap1 and Nrf2 and downstream HO-1 were examined using qPCR after Nestin knockdown. The results showed that Nestin knockdown did not change the mRNA expression level of Keap1 and Nrf2; however, the HO-1 mRNA expression level was significantly decreased following Nestin knockdown (Figs. 5A–C). In addition, Nestin knockdown showed no effect on Keap1 protein expression level; however, both Nrf2 and HO-1 protein expression levels were downregulated after Nestin knockdown (Fig. 5D, E). Keap1 is a substrate adaptor that carries Nrf2 to the E3 ubiquitin ligase complex, leading to rapid proteasome-mediated Nrf2 degradation [21]. Because Nestin knockdown did not influence Keap1 expression, we explored whether Nestin prevents Nrf2 degradation through its interaction with Keap1. The immunoprecipitation assay specifically indicated that Nestin could directly bind with Keap1 (Fig. 5F). We next speculated whether Nestin modulates the expression levels of antioxidant molecules through the Nrf2–ARE pathway. To confirm this speculation, SGC-7901 and MKN-45 cells were exposed to the ARE luciferase reporter. The results showed that Nestin knockdown obviously inhibited luciferase reporter activity (Fig. 5G). These observations indicated that Nestin silencing downregulated the signal transduction of Keap1–Nrf2–HO-1 axis, probably through Keap1 binding.

### Nrf2 participated in Nestin-modulated tumor phenotypes and the antioxidant capacity of GC cells

**Fig. 6:**
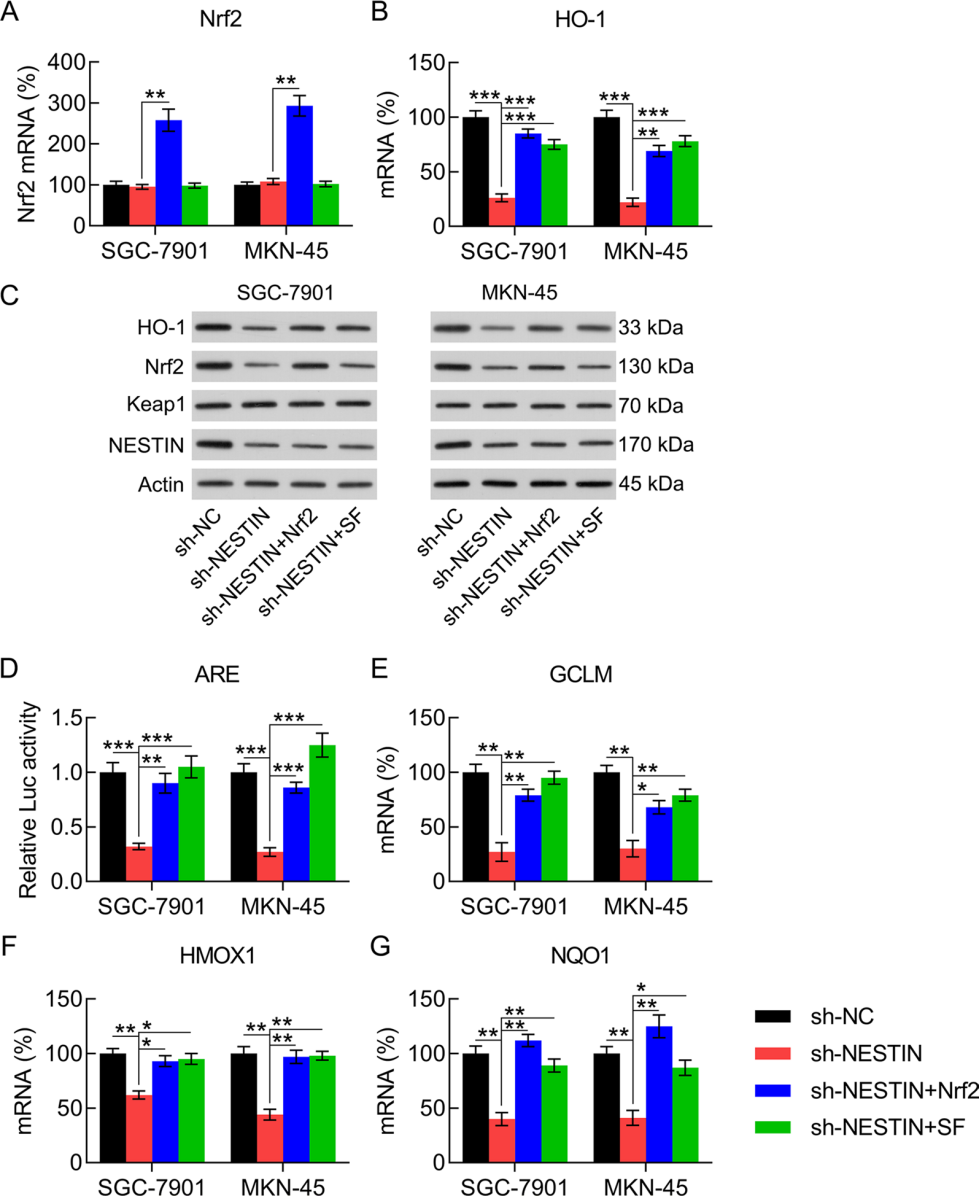
Nrf2 overexpression and SF treatment in GC cells. GC cells with Nestin knockdown were transfected with Nrf2-overexpressing vector for 36 h or treated with 10 μM SF for 36 h. **A**, **B** qPCR revealed the effect of Nestin knockdown on the mRNA expression levels of Nrf2 and HO-1 in GC cells. **C** WB revealed the effect of Nestin knockdown on the protein expression levels of Nrf2 and HO-1 in GC cells. **D** GC cells with Nestin knockdown were transfected with ARE luciferase reporter. The luciferase activity was determined 1.5 d after transfection. **E**–**G** qPCR revealed the effect of Nestin knockdown on the expression levels of the antioxidant-related genes *GCLM*, *HMOX1*, and *NQO1* in GC cells (n = 3). The results are expressed as means ± SD. *P < 0.05, **P < 0.01, ***P < 0.001 vs. the indicated groups

As Nrf2 expression is repressed after Nestin knockdown, we determined whether Nrf2 is associated with Nestin-mediated tumor phenotypes and the antioxidant capacity of GC cells. GC cells with Nestin knockdown were cotransfected with an Nrf2-overexpressing vector or the Nrf2 activator sulforaphane (SF) [22] to upregulate Nrf2 expression or activity, respectively. qPCR confirmed that the Nrf2 expression level was significantly improved following the transfection of Nrf2-overexpressing vector in all cells (Fig. 6A). The downstream HO-1 mRNA expression was also upregulated after Nrf2 overexpression or SF treatment (Fig. 6B). Furthermore, WB analysis showed a trend similar to the qPCR results (Fig. 6C). Furthermore, Nrf2 overexpression and SF treatment enhanced the ARE luciferase reporter activity and mRNA expression levels of *GCLM*, *HMOX1*, and *NQO1* (Fig. 6D–G), which suggests that Nrf2 overexpression and SF treatment activates Nrf2–ARE signal transduction in GC cells.

### Nrf2 overexpression and SF treatment counteracted the effect of Nestin knockdown on tumor phenotypes and the antioxidant capacity of GC cells

**Fig. 7:**
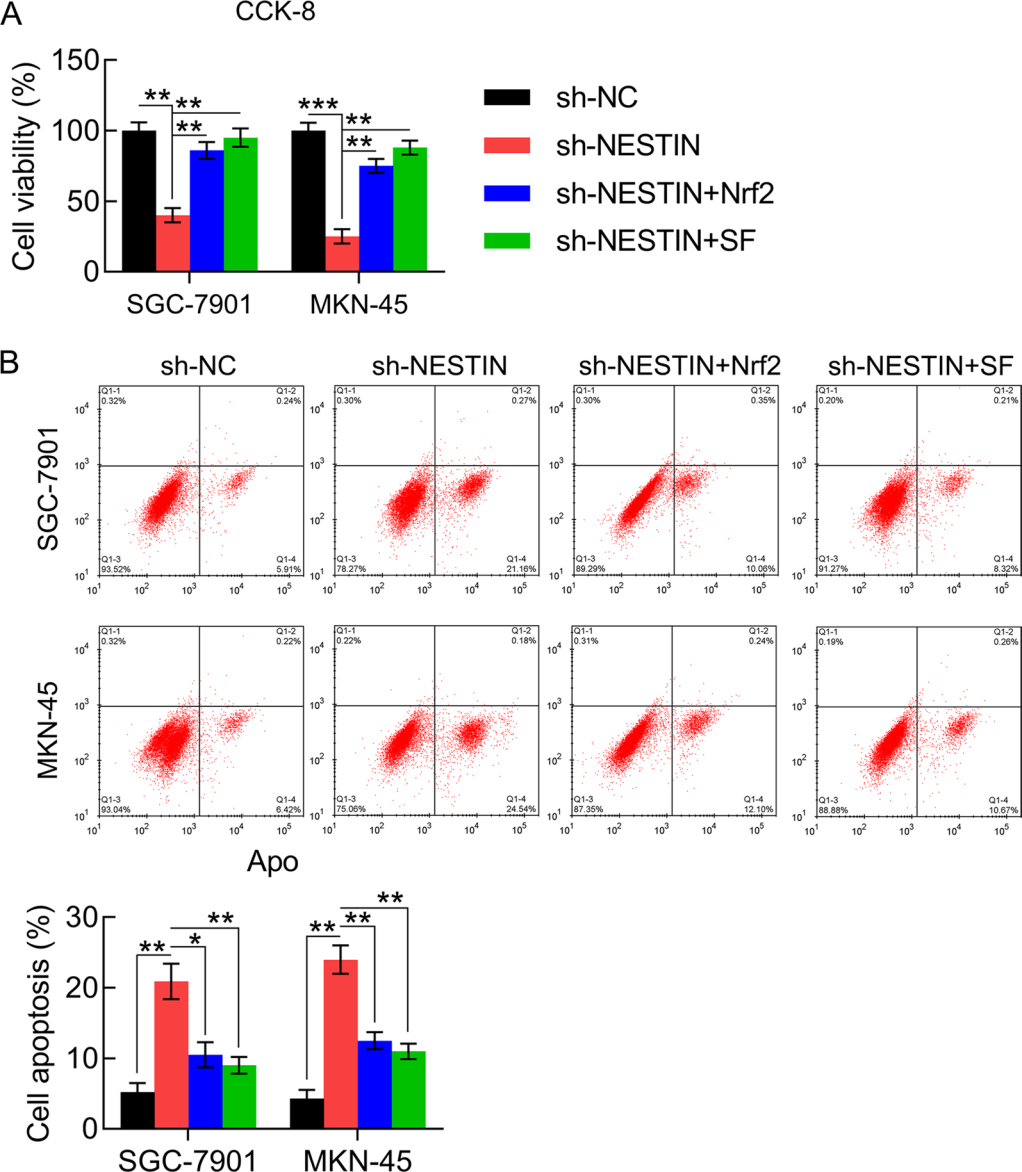
Effect of Nrf2 overexpression and SF treatment on cell viability and apoptosis in Nestin-depleted GC cells. GC cells with Nestin knockdown were transfected with Nrf2-overexpressing vector for 36 h or treated with 10 μM SF for 36 h. **A** The CCK-8 assay revealed the viability of GC cells 2 d after transfection. **B** Annexin V-FITC/PI FC revealed the percentage of apoptotic cells (n = 3). The results are expressed as means ± SD. *P < 0.05, **P < 0.01, ***P < 0.001 vs. the indicated groups

Given the previous study results indicating that Nestin and Nrf2 play essential roles in tumor phenotype and cellular redox homeostasis of lung cancer, we hypothesized that Nrf2 is also involved in the Nestin-modulated GC cell viability, apoptosis, antioxidant gene expression, and metastasis. The CCK-8 assay and Annexin V-FITC/PI FC were performed to evaluate the role of Nrf2 overexpression and SF treatment on Nestin-modulated cell viability and apoptosis. Compared with the single Nestin silencing group, cell viability was clearly increased in GC cells with Nrf2 overexpression and SF treatment (Fig. 7A, B).

**Fig. 8:**
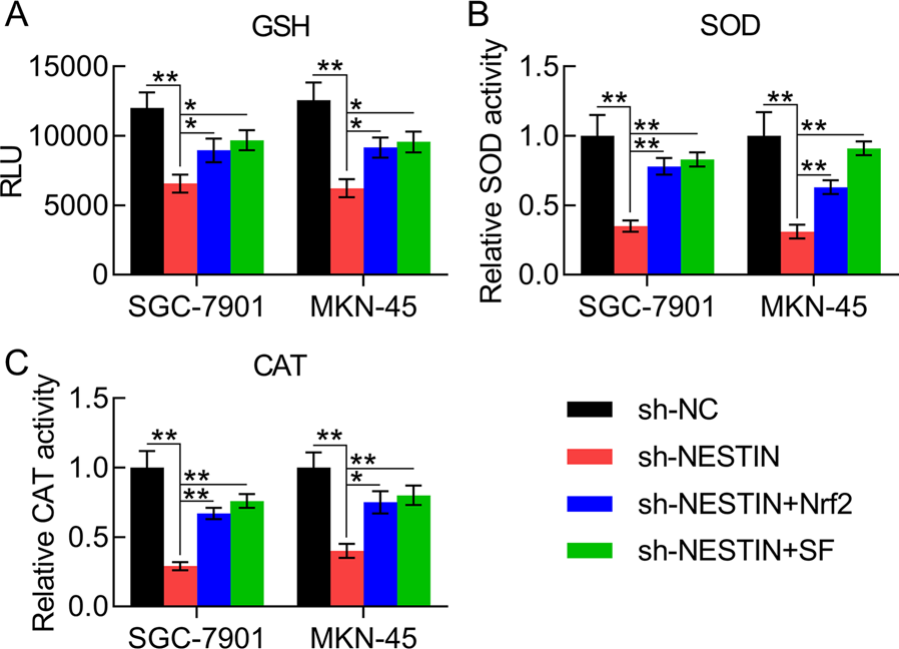
Effect of Nrf2 overexpression and SF treatment on the antioxidant capacity of Nestin-knockdown GC cells. GC cells with Nestin knockdown were transfected with Nrf2-overexpressing vector for 36 h or treated with 10 μM SF for 36 h. **A**–**C** GSH levels, SOD activity, and CAT levels were examined in GC cells (n = 3). The results are expressed as means ± SD. *P < 0.05, **P < 0.01 vs. the indicated groups

To further determine whether Nrf2 participated in Nestin-associated antioxidant capacity, SOD activity, GSH level, and CAT activity were examined. The three indices were partially restored following Nrf2 overexpression or SF treatment (Fig. 8A–C), suggesting that Nrf2 is involved in Nestin-related antioxidant capacity.

**Fig. 9:**
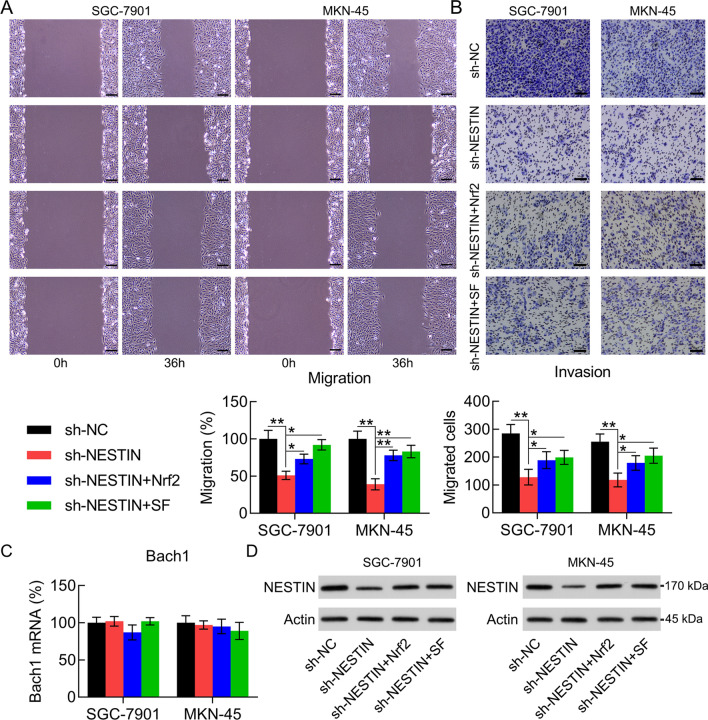
Effect of Nrf2 overexpression and SF treatment on the migration and invasion abilities of Nestin-knockdown GC cells. GC cells with Nestin knockdown were transfected with Nrf2-overexpressing vector for 36 h or treated with 10 μM SF for 36 h. **A** The migration and **B** invasion abilities of GC cells were assessed using wound healing and Transwell invasion assays. Scale bar, 50 μm. **C** qPCR and **D** WB analysis were used to determine the mRNA and protein expression levels of Bach1, respectively, in GC cells (n = 3). The results are expressed as means ± SD. *P < 0.05, **P < 0.01 vs. the indicated groups

To examine whether Nrf2 regulates Nestin knockdown-repressed GC cell metastasis, the wound healing assay and Transwell invasion assay were performed and the Bach1 expression level was determined using both qPCR and WB analysis. The results showed that Nrf2 overexpression and SF treatment resulted in the recovery of the migration and invasion ability of GC cells (Fig. 9A, B). In addition, qPCR results revealed that Nrf2 upregulation or activation did not affect the Bach1 mRNA expression level. However, WB images showed that the Bach1 protein expression level was upregulated following Nrf2 restoration (Fig. 9C, D).

### Nestin knockdown inhibited tumor growth of GC in vivo

**Fig. 10:**
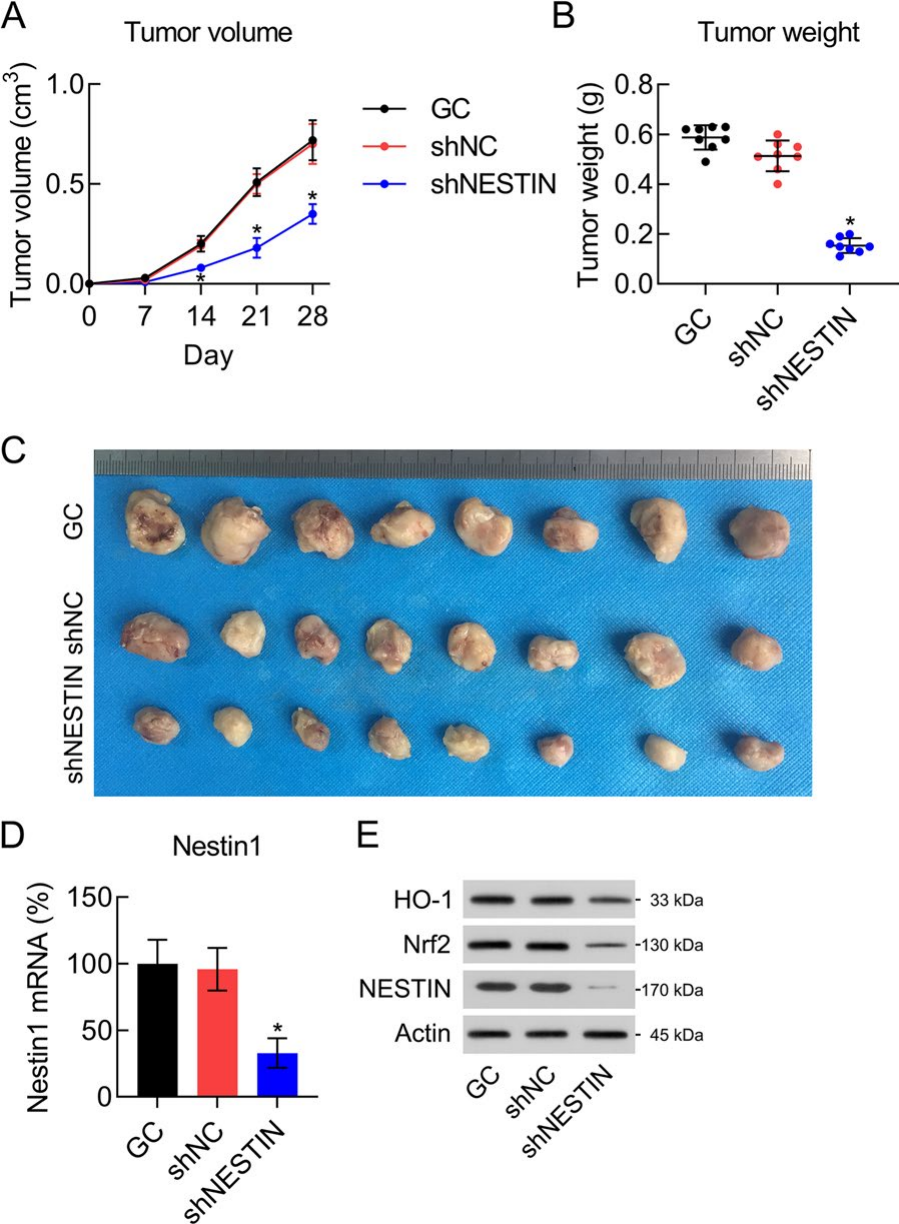
Nestin knockdown inhibits xenograft GC tumorigenesis. The right back flank of 6-week-old BALB/c-nu mice (n = 8/group) were transfected with the subcutaneous injections of SGC-7901, SGC-7901-sh-NC, and SGC-7901-sh-SNHG11 cells. **A** The tumor size (growth rate) was determined over time. **B** Tumors were harvested 4 weeks after grafting and weighed. **C** Images of all tumors. **D**, **E** The mRNA and protein expression levels of Nestin in the tumor samples were determined using WB and qPCR, respectively, (n = 8). The results are expressed as means ± SD. *P < 0.05, **P < 0.01 vs. the indicated groups

The mice were subcutaneously injected with SGC-7901, SGC-7901-sh-NC, and SGC-7901-sh-Nestin cells to investigate the role of Nestin using a xenograft of GC tumor growth. Tumor volume monitoring revealed that Nestin knockdown slowed the tumor growth rate (Fig. 10A). Mice were euthanized at day 28, and tumors was removed and weighed. Tumors in the sh-NC groups had a higher mean tumor weight than those in the Nestin knockdown group, (Fig. 10B, C). The expression level of Nestin in the tumor specimens (n = 8) of mice in each group was also examined. Nestin mRNA expression appeared to be downregulated in the SGC-7901-sh-Nestin inoculated mice compared with that in mice in the sh-NC-inoculated groups (Fig. 10D, E).

## Discussion

Intracellular oxidative stress modulation is considered an effective cancer treatment. The present study confirmed that Nestin was overexpressed in GC tissues and cells and bound to Keap1 to stabilize Nrf2 expression, thereby promoting cell viability, preventing cell apoptosis, increasing antioxidant capacity, and maintaining metastasis. We also showed that Nrf2 overexpression or SF treatment in GC cells counteracted the effect of Nestin knockdown on tumor phenotypes and antioxidant capacity. Overall, these results indicate that the Nestin–Keap1–Nrf2 axis is associated with the antioxidant responses and redox homeostasis of GC cells.

Nestin is a common marker of multipotent stem cells [23], and its expression is significantly upregulated in tissue injury and cancer progression [24]. Previous reports have provided insights into the molecular mechanisms of Nestin in tumor development. Progenitor cells positive for Nestin in the cerebellum displayed more effective tumor cell transformation and severe genomic instability compared with cells that were negative for Nestin [25]. Nestin-expressing progenitor-like cells that are dedifferentiated from mature hepatocytes evolve into cholangiocarcinomas or hepatocellular carcinomas [26]. siRNA has been shown to exert a tumor-suppressive effect in vivo by inhibiting tumor angiogenesis [27], suggesting that Nestin is a novel treatment target for many types of tumors. The present study reported that Nestin played a vital role in maintaining the GC cell redox balance. Meanwhile, Nestin expression was closely associated with malignancies, including tumor proliferation and metastasis. An in vivo tumorigenesis experiment further demonstrated the influential role of Nestin during tumor growth, which indicates the mechanisms underlying Nestin modulation and exertion of antioxidant effects as well as the potential treatment targets that may be used to prevent tumor growth.

Oxidative stress was involved in the progression of GC by affecting the expression of critical effectors [10]. Nrf2 is a well-documented antioxidative regulator that upregulates the expression levels of various antioxidant enzymes, including glutathione S-transferase and NQO1, by binding with ARE enhancer sequences. An ectopic increase in the Nrf2 expression level has been detected in various cancers, including breast, pancreatic, and head and neck cancers. Patients with high Nrf2 expression level usually exhibited an unfavorable prognosis [28, 29]. A high Nrf2 expression level was associated with both antioxidant response and metabolism reprogramming. In lung cancer cells, Nrf2 redirects glutamine and glucose metabolism to anabolic pathways via metabolic reprogramming, which benefits uncontrolled cancer cell proliferation [30]. Nrf2 also regulates aerobic glycolysis via HIF-1α in breast cancer [31]. In the present study, the stabilization of Nrf2 expression was promoted by Nestin, as evidenced by Nestin knockdown, which led to the Nrf2 protein expression level being downregulated rather than the mRNA expression level. Moreover, Nestin knockdown attenuated Nrf2-mediated redox homeostasis and antioxidant gene expression by directly binding with Keap1. Nrf2 also participated in Nestin-modulated cell viability and proliferation and played an important role in GC cell migration and invasion, as previously reported [20]. Taken together, these results improve our knowledge of the mechanism by which Nrf2 activation regulates Nestin in GC cells.

## Conclusions

In conclusion, this study confirmed the interaction between the Keap1–Nrf2 axis and Nestin and that Nestin can mediate antioxidant responses and maintain tumor phenotypes in GC. Further, the results revealed that the association among the Nestin–Keap1–Nrf2 pathway, tumor phenotypes, and antioxidant defenses could be regarded as a possible treatment target for GC.

## Data Availability

The manuscript has no associated data.

## Abbreviations

GC: Gastric cancer
Keap1: Kelch-like ECH-associated protein 1
Nrf2: Nuclear factor erythroid 2-related factor 2
AREs: Antioxidant response elements
Cul3: Cullin 3
qPCR: Quantitative polymerase chain reaction
WB: Western blot
CCK-8: Cell Counting Kit-8
BrdU: Bromodeoxyuridine
FC: Flow cytometry
GSH: Glutathione
SOD: Superoxide dismutase
GCL: Glutamate–cysteine ligase
GCLM: GCL modifier subunit
SOD1: Superoxide dismutase 1
GPX1: Glutathione peroxidase 1
CAT: Catalase
SF: Sulforaphane
